# Direct Analyses of Secondary Metabolites by Mass Spectrometry Imaging (MSI) from Sunflower (*Helianthus annuus* L.) Trichomes

**DOI:** 10.3390/molecules22050774

**Published:** 2017-05-10

**Authors:** Denise Brentan Silva, Anna-Katharina Aschenbrenner, Norberto Peporine Lopes, Otmar Spring

**Affiliations:** 1 Laboratório de Produtos Naturais e Espectrometria de Massas (LaPNEM), Universidade Federal de Mato Grosso do Sul, Campo Grande 79070-900, MS, Brazil; denise.brentan@ufms.br; 2 Núcleo de Pesquisas em Produtos Naturais e Sintéticos (NPPNS), Faculdade de Ciências Farmacêuticas de Ribeirão Preto, Universidade de São Paulo, Ribeirão Preto 14040-020, SP, Brazil; 3 Institute of Botany, University of Hohenheim, Garbenstraße 30, Stuttgart 70593, Germany; katharina.aschenbrenner@uni-hohenheim.de

**Keywords:** MALDI, LDI, trichome, polymethoxylated flavonoids, *Helianthus*, mass spectrometry, imaging, sesquiterpene lactones, sesquiterpenes

## Abstract

*Helianthus annuus* (sunflower) displays non-glandular trichomes (NGT), capitate glandular trichomes (CGT), and linear glandular trichomes (LGT), which reveal different chemical compositions and locations in different plant tissues. With matrix-assisted laser desorption/ionization (MALDI) and laser desorption/ionization (LDI) mass spectrometry imaging (MSI) techniques, efficient methods were developed to analyze the tissue distribution of secondary metabolites (flavonoids and sesquiterpenes) and proteins inside of trichomes. Herein, we analyzed sesquiterpene lactones, present in CGT, from leaf transversal sections using the matrix 2,5-dihydroxybenzoic acid (DHB) and α-cyano-4-hydroxycinnamic acid (CHCA) (mixture 1:1) with sodium ions added to increase the ionization in positive ion mode. The results observed for sesquiterpenes and polymethoxylated flavones from LGT were similar. However, upon desiccation, LGT changed their shape in the ionization source, complicating analyses by MSI mainly after matrix application. An alternative method could be applied to LGT regions by employing LDI (without matrix) in negative ion mode. The polymethoxylated flavones were easily ionized by LDI, producing images with higher resolution, but the sesquiterpenes were not observed in spectra. Thus, the application and viability of MALDI imaging for the analyses of protein and secondary metabolites inside trichomes were confirmed, highlighting the importance of optimization parameters.

## 1. Introduction

Trichomes are specialized structures distributed on the surface of many plants, and are specifically expressed on different organs with a wide variety of shapes [1]. Non-glandular trichomes (NGT) show functions which may be related to heat reduction, to increase water absorption or drought tolerance, resist freezing or protect from UV radiation and insect predation [2]. Recently, even their involvement in petiole movement has been described. Hence, these plant hairs act as a biomechanical system and reservoir of hydrostatic pressure [3].

Contrarily, glandular trichomes are mainly related to defense against pathogens and herbivores, producing various allelochemicals such as terpenoids, phenylpropenoids, methyl ketones, acyl sugars, flavonoids, and defensive proteins [4,5,6,7,8]. Easily accessible at the plant surface, glandular trichomes are good models for the study of biochemical pathways as they facilitate the determination of genes involved in these pathways, as was shown for monoterpenes [5], sesquiterpenes [9] or sesquiterpene lactones [10,11,12,13], and stilbenes [14]. Thus, such results from glandular trichomes may have high economic relevance and future applications in the biotechnological production of flavoring substances and pharmaceuticals [1,15], or in plant breeding for pest resistance [14,16].

Current trichome metabolite analyses are typically performed through mechanical harvest [1,5,17] and extracting compounds with organic solvent for subsequent chromatographic purification and spectrometric measurements (MS and NMR). Online techniques, such as LC-MS, GC-MS, or LC-NMR [18], can facilitate this process, but the sampling of trichomes to establish composition at the individual level is still a challenge [19]. Additional methods to identify and visualize the location of specific target compounds or the expression of key enzymes involved in their production would be highly desirable. In this context, mass spectrometry imaging (MSI) has recently been applied to determine the exact individual constituents inside trichomes [20]. The trichomes of tomato leaves were printed on a carbon layer and analyzed by laser desorption/ionization (LDI), annotating acyl sugars, an alkaloid, and flavonoids [21]. LDI was also applied to identify flavonoids and naphthoquinones from glandular trichomes of *Hypericum perforatum* [22], while MALDI imaging was used to determine the presence of sesquiterpene lactones in glandular trichomes of leaves from *Smallanthus sonchifolius* [23]. However, the inhomogeneity of surface trichome tissues directly influences the quality of the results in MSI, creating some issues in correctly analyzing these trichomes [20,23], such as the fragility, different distances between trichomes on the surface, and different morphological characteristics [21]. Although MSI is an innovative and powerful technique, it has still not been widely explored for trichome analyses and more applied studies are necessary to improve its broader application. For that reason, we decided to use the sunflower, a well-studied model plant with trichome metabolism, to explore the possibilities and limitations of MSI analysis on trichome metabolites and proteins.

**Figure 1:**
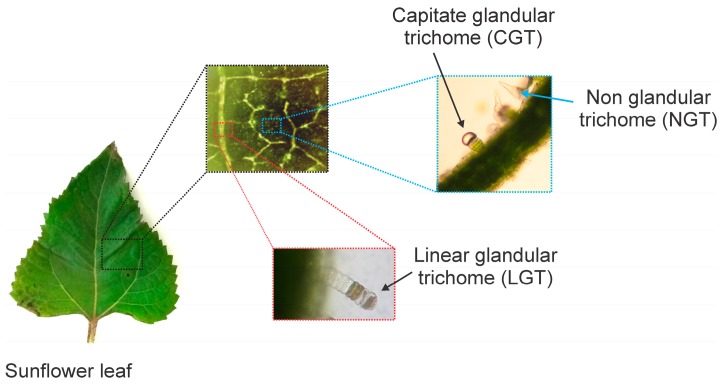
Sunflower leaf with non-glandular trichomes (NGT), capitate glandular trichomes (CGT) in intercostal areas, and linear glandular trichomes (LGT) along leaf veins.

Non-glandular trichomes (NGT), capitate glandular trichomes (CGT), and linear glandular trichomes (LGT) (Figure 1) have been described from *Helianthus annuus* L. (Asteraceae) [17]. They present differences in cellular structure, location on plant surface, cytological development [24,25,26,27], and chemical constituents [28,29,30,31]. Besides their metabolic profiles, the enzymes involved in the biosynthesis of sesquiterpene lactones (STL) from CGT were recently revealed when three sesquiterpene synthases [10,11] and various cytochrome P450 enzymes from their stalk cells were characterized [12,13]. Similarly, LGT located on stems, leaf veins, and involucral bracts [32] accumulate bisabolene type sesquiterpenes along with polymethoxylated flavonoids [29,30], and expression studies revealed the presence of transcripts for phenylalanine lyase (HaPAL), chalcone synthase (HaCHS), farnesylpyrophosphate synthase (HaFPPS), and bisabolene synthase (HaTPS12) in the secretory active trichome stage [9].

In this study, we present a successful method for selective MALDI and LDI imaging of trichomes from *H. annuus* leaves, avoiding the complicated isolation by microsampling. We aim to detect the different secondary metabolites (sesquiterpenes, STL, and flavonoids) and proteins by MSI through the development of sensitive methods capable of profiling particular compounds in trichomes with high spatial resolution.

## 2. Results and Discussion

### 2.1. Verification of Target Compounds by Ultra-Performance Liquid Chromatography with Diode Array Dectection and Mass Spectrometry (UPLC-DAD-MS)

**Figure 2:**
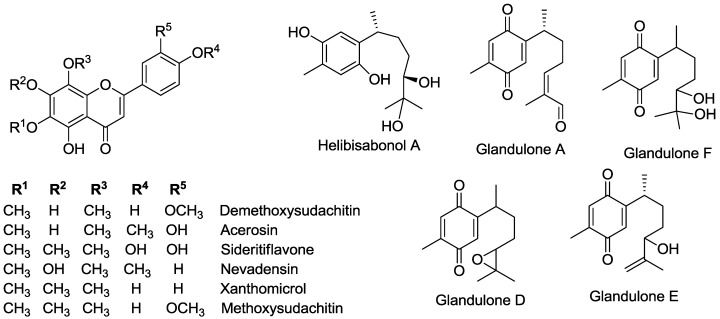
Structure of polymethoxylated flavones and sesquiterpenes identified from LGT.

To verify the occurrence of LGT- and CGT-specific metabolites known from previous reports in the plant material used for MSI, trichome extracts were prepared and analyzed by UPLC-DAD-MS (data in the Supplementary Material) and MALDI-MS experiments (item 2.2). LGT collected manually from the central rib vein of mature sunflower leaves presented the known polymethoxylated flavones and sesquiterpenes [29,31]. Metabolite identification was performed by the injection of authentic standards and from UV and MS data compared with previous reports [29,33,34], thus confirming the presence of the polymethoxylated flavones demethoxysudachitin, acerosin, sideritiflavone, nevadensin, xanthomicrol, and methylsudachitin (Figure 2, Figure S1 and Table S1—Supplementary Material). In addition, the data confirmed the presence of the sesquiterpenes helibisabonol A (C_15_H_24_O_4_, MW: 268), glandulone A (C_15_H_18_O_3_, MW: 246), glandulone F (C_15_H_22_O_4_, MW: 266), glandulone D (C_15_H_20_O_3_, MW: 248) and glandulone E (C_15_H_20_O_3_, MW: 248). The three compounds helibisabonol C, heliannuol A, and heliannuol D (all with the same molecular weight of C_15_H_22_O_3_, MW: 250) could not be differentiated, since only one chromatographic peak showed the expected ion at *m*/*z* 251 and the fragment ions observed were not sufficient to characterize the chemical structure.

### 2.2. Optimization and MALDI/LDI-MSI

**Figure 3:**
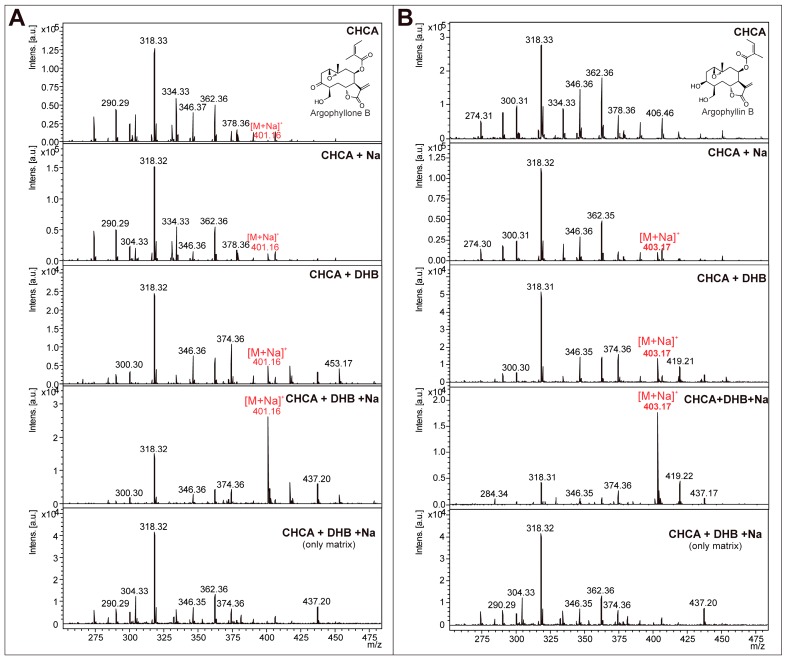
MALDI mass spectra (positive ion mode) of sesquiterpene lactone argophyllone B (MW: 378, **A**) and argophyllin B (MW: 380, **B**) with different matrix compositions and mass spectra of the matrices.

With respect to the STL from CGT of sunflower leaves, argophyllone B and argophyllin B were already described compounds [31], but previous investigations showed that STL were not very suitable for MALDI due to the insufficient ionization and/or intense fragmentation in the source [35], requiring an optimization of the MALDI-MS method for application in MSI. The ionization of the standards argophyllone B and argophyllin B were possible using the matrices 2,5-dihydroxybenzoic acid (DHB) and α-cyano-4-hydroxycinnamic acid (CHCA), but only the sodiated ions were observed (Figure 3). The best result was achieved with the addition of sodium salts together with a 1:1 mixture of DHB:CHCA, increasing sodiated ions at *m*/*z* 403 and 401 relative to argophyillin B and argophyllone B, respectively (Figure 3). Recently, a similar effect was found for uvedalin and enhydrin, two compounds from *Smallanthus sonchifolius* [23].

**Figure 4:**
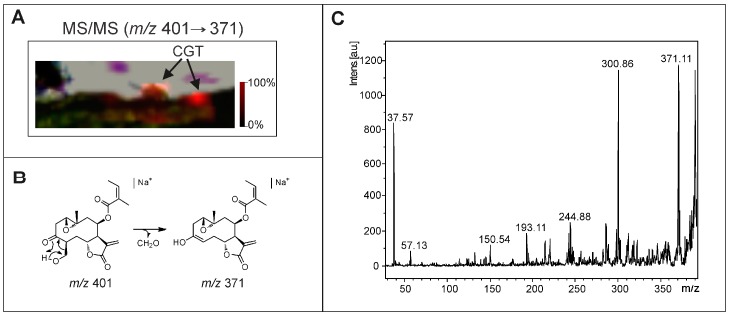
MALDI-MS/MS image reconstructed with the fragment ion *m*/*z* 371 obtained from sodiated ion *m*/*z* 401 (argophyllone B) (**A**) (the arrows indicate the region of capitate glandular trichomes, CGT), its fragmentation pathway (**B**), and the MS/MS spectrum of argophyllone B (*m*/*z* 401 [M + Na]^+^) (**C**).

**Figure 5:**
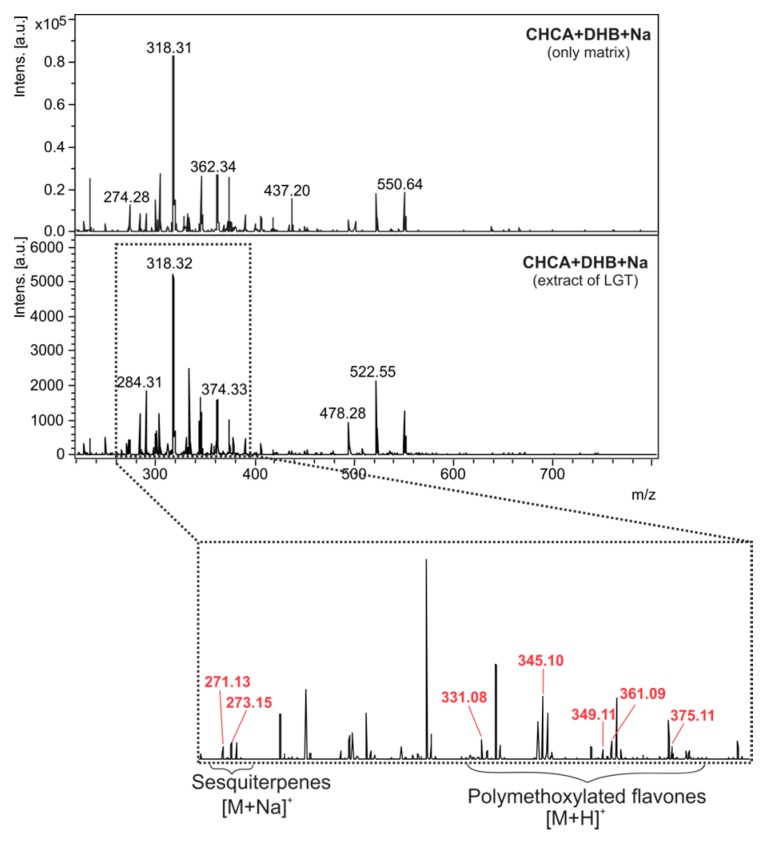
MALDI mass spectra (positive ion mode) of the matrix and linear glandular trichome (LGT) extract (the ions of its constituents are highlighted in red).

Therefore, the CGT of sunflower were analyzed by MALDI-MSI using the matrices CHCA and DHB with the addition of sodium salt. However, several difficulties were found due to the small size of these trichomes, requiring care to locate them. The ion map distribution from transversal leaf sections was constructed based on the ions *m*/*z* 403.17 and 401.16 [M + Na]^+^, which correspond to argophyllin B and argophyllone B (Figure S2, Supplementary Material), respectively, and the results indicated the presence of them (Figure 4) in CGT. Moreover, the images were also constructed from MALDI-MS/MS data, for example, using the fragment ion (*m*/*z* 371) of ion *m*/*z* 401 that was applied to confirm their location on CGT (Figure 5). Thus, the results demonstrate the huge potential of MALDI-MSI to analyze the metabolites inside of trichomes, even small ones. The fragment ions at *m*/*z* 371 and 301 are yielded by the loss of CH_2_O (formaldehyde), an angelate substituent, and a water molecule; both reconstructed images from them revealed the same ion distribution.

**Figure 6:**
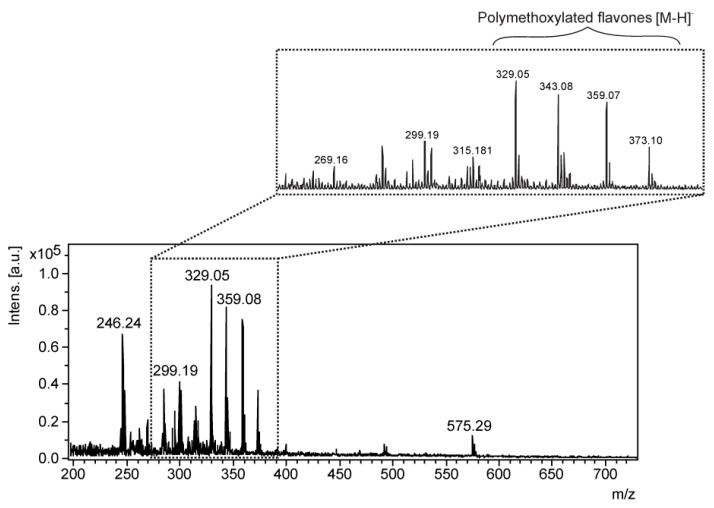
LDI mass spectra (negative ion mode) of linear glandular trichome (LGT) extract (without a matrix).

On the leaves of *H. annuus*, LGT are located close to the leaf veins, while CGT are observed on intercostal areas [32,36]. Thus, the MALDI-MS spectrum (matrix DHB:CHCA (1:1) added sodium, positive ion mode) of the extracts from the central rib epidermis revealed the sodiated ions *m*/*z* 271.1 and 273.1 (Figure 5) that correspond to the compounds glandulone C/D/E and helibisabonol C/heliannuol A or D, respectively. In addition, even without a matrix, the polymethoxylated flavones revealed lower intensity in these spectra in comparison to the negative ion mode spectra (Figure 6). Furthermore, when the matrix was added, some of them showed ions with *m*/*z* close to matrix ions, requiring careful evaluation due to the inhomogeneity of tissue and indicating that an internal calibration is highly recommended.

Another important point is the absence of ions from sesquiterpenes in negative ion mode spectra. Thus, an efficient method for mass spectrometry imaging (MSI) could be carried out through positive ion mode using the mixture of DHB and CHCA matrices with the addition of sodium ions, as evaluated here and described by Lopes and collaborators [23]. So, both compounds (flavonoids and sesquiterpenes) can be ionized and observed in the spectra of extracts and MSI experiments. However, the matrix application revealed problems for MSI in our studies, since the size of sunflower trichomes is very small (only visible through a microscope) [32] and LGT shrink and change shape during the dehydration process, caused by the vacuum of the ionization source and the steps of sample preparation. Therefore, we observed difficulties in obtaining reliable results and resolution of the images in MSI. Such issues, related to the dehydration of in natura plant tissue for MALDI-MSI, have been acknowledged and discussed in review articles [20,35,37]. The small size of LGT allows only the existence of very small matrix crystals which must be homogeneously distributed in the tissue, and these requirements were impracticable and difficult to achieve due to the dehydration of tissue. Likewise, the application of the matrix can relocate the analytes [38,39], requiring an evaluation of this effect. Spatial resolution is essential to confirm and determine the constituents that are inside LGT, which is related to the size of the laser focus and matrix crystals in the tissues [20].

Therefore, an alternative method was acquired by LDI-MSI, since polymethoxylated flavones are compounds previously reported in LGT of sunflowers cultivated in Europe [29]. Data obtained by UPLC-MS also confirmed the production and accumulation of such compounds in the cultivars of Brazil (item 1.2, Supplementary Material). In addition, the LDI spectrum of LGT extract (Figure 6) confirmed the accumulation of these polymethoxylated flavones. Thus, MSI experiments were performed without a matrix due to the auto-ionization of these flavonoids, which can act similarly to a matrix and ionize other compounds from a sample, as recently demonstrated [40].

**Figure 7:**
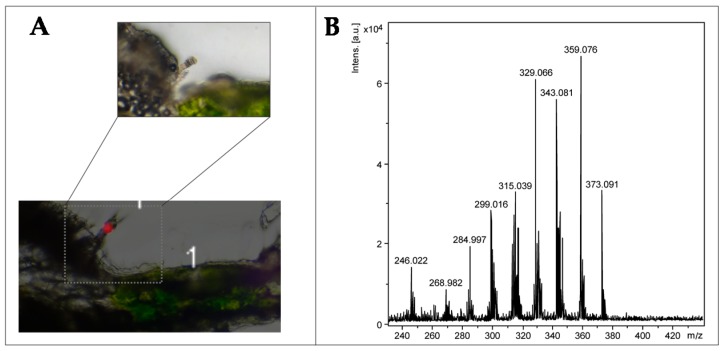
LDI-MS image reconstructed from the ions *m/z* 329.06, 343.08, 359.08 and 373.09 [M − H]^−^, highlighting the linear glandular trichome (LGT) (**A**) and mass spectrum from LDI-MSI (**B**).

**Figure 8:**
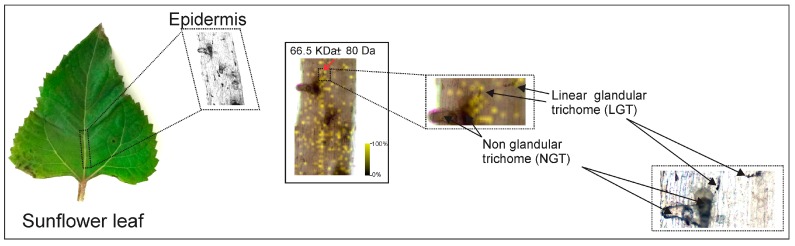
MALDI-MS image reconstructed with the ion *m*/*z* 66,500 (positive ion mode) from the central rib epidermis of *H. annuus*.

The transversal sections of sunflower leaves produce isolated regions with trichomes and therefore were applied for MSI analyses, resulting in a more reliable ion map distribution since there was no overlap of tissues. Thus, the transversal sections were analyzed by LDI-MSI using negative ion mode, and the images were reconstructed from the ions *m*/*z* 329.06, 343.08, 359.08, and 373.09 [M − H]^−^ (Figure 7 and Figure S3, Supporting Material), which are correlated to the polymethoxylated flavones 1–6, confirming their location in LGT. The sesquiterpenes, previously identified by UPLC-DAD-MS (item 1.2, Supplementary Material), were not observed in these analyses without a matrix and in negative ion mode. However, a protein at 66.5 KDa was detected by MALDI-MSI from the central rib epidermis in the region of LGT, and was tentatively assigned to the bisabolene synthases (two isoforms), related to the biosynthesis of sesquiterpenes (Figure 8). These bisabolene synthases were previously isolated from *Helianthus annuus*, and they are presumably involved in the biosynthesis of sesquiterpenes through the cyclization of farnesyl-pyrophosphate to (*Z*)-*γ*-bisabolene [9,29]. Thus, these data suggest that the technique can be applied to locate and detect enzymes related to the biosynthesis of metabolites on trichomes, however, the spatial resolutions were lower than those observed for images from the polymethoxylated flavones because the matrix was essential for the ionization of proteins.

These results demonstrated the efficiency and applicability of MALDI or LDI imaging for trichome analyses. However, it is necessary to consider the characteristics of the trichome (size and shape) and the target metabolites when developing such methods. Although these methodologies have been applied in several studies, analyses of trichomes are scarce. Herein, the advantages of these methods were proven in the direct profile of secondary metabolites, mainly for highly conjugated compounds (flavonoids), since a matrix was not required, and thus several problems related to matrix application (isobaric ions, detector saturation, delocation of metabolites) were avoided.

Through the results of ion map distribution, MSI may also be used to hypothesize about and understand ecological interactions [20]. In plants, defense compounds are shown to accumulate on specialized structures with a single or few cells, such as glandular trichomes. Meanwhile, non-glandular trichomes can also represent an additional defense mechanism due to their role in the reduction of plant accessibility [41,42]. Furthermore, knowledge about the chemical composition of trichomes can elucidate such putative defense mechanisms.

Different chemical compositions and locations on leaves have been described for CGT and LGT [28,29,30,32,36]. CGT are distributed on intercostal areas, and show a multicellular basis and a capitate-like cavity where sesquiterpene lactone angelates (such as argophyllin B and argophyllone B) are accumulated, and exert high feeding deterrence [30,32,36,43]. LGT are distributed along leaf veins and the petiole, which are formed by linear rows with six to ten cells, and their chemical composition reveals the presence of sesquiterpenes (such as glandulones, helibisabonols and heliannuols) and polymethoxylated flavones [29,32]. The flavonoids, including nevadensin, exhibit poor antifeedant properties [43], suggesting that the function of polymethoxylated flavones may be different in relation to sesquiterpene lactones, probably being related to UV protection [44,45]. Meanwhile, the antifeedant sesquiterpene lactones are located on intercostal areas (inside of CGT) to protect against herbivores, since these tissue parts are softer than vessel areas and are preferred by herbivores due to their higher digestibility [42,44]. Therefore, trichomes with different chemical compositions and locations on different plant tissues may explain the defense strategies of plants.

## 3. Materials and Methods

### 3.1. Plant Material

*Helianthus annuus* cv. Giganteus plants were cultivated in pots and kept outdoors until flowers developed. Leaf tissue was collected and used directly for MALDI imaging or air dried to collect trichomes used in UPLC-DAD-MS experiments.

### 3.2. Ultra-Performance Liquid Chromatography with Diode Array Dectection and Mass Spectrometry (UPLC-DAD-MS) Analyses

Plant trichomes were manually collected for analysis by UPLC-DAD-MS (ACQUITY UPLC-MS System, Waters Assoc., Milford, MA, USA) in order to validate the vegetal material. Acetonitrile and deionized water (9:1, *v*/*v*) were added to the collected linear glandular trichomes (ca. 100 per sample), and maintained for 8 min in an ultrasonic bath with 200 μL of solvent (acetonitrile and water 1:1, *v*/*v*). Subsequently, samples were centrifuged and filtered through a 13-mm PTFE membrane (0.22 mm pore size, Millex, Millipore, Darmstadt, German). The UPLC—DAD-MS System was used with a chromatographic column ACQUITY 1.7 mm C18 BEH column (2.1 mm, 50 mm, Waters Assoc., Milford, CT, USA) at 30 °C. Acetonitrile (B) and deionized water (A), both containing 0.1% formic acid, were used as a mobile phase, applying a flow rate of 0.3 mL/min and the following elution profile: 0 to 11 min, 20 to 100% B; 11 to 13 min, 100% B; 13 to 13.5 min, 100 to 20% B; and 13.5 to 17 min 20% B (column equilibration). The injection volume was 8 μL and the samples were conditioned in the automatic injector at 10 °C. The mass spectrometer was provided as an electrospray ionization source coupled with a triple quadrupole analyzer. The following parameters were applied in the analyses: capillary energy of 2.4 kV, cone voltage of 15.0, source temperature of 150 °C, desolvation temperature and flow of 350 °C and 650 L/h, respectively. Nitrogen was used as the nebulizing and drying gas, and argon was used as the collision gas. All samples were analyzed in negative and positive ion modes. The secondary metabolites were identified by retention time, UV, and MS data, and compared with published data for *H. annuus* [29]. The flavonoid xanthomicrol was confirmed by co-injection with an authentic sunflower reference sample [29]. This was also the case for the STL argophyllin B and argophyllone B, which were confirmed in a recent study on the metabolome of sunflower glandular trichomes [31].

### 3.3. MALDI-MS and LDI-MS of LGT Extracts and Standards

The standards (xanthomicrol, argophyllin B and argophyllone B) and LGT extract (collected manually) were analyzed by MALDI and/or LDI-MS and MS/MS. A MALDI-TOF/TOF UltrafleXtreme (Bruker Daltonics, Bremen, Germany) was used for these analyses. The matrices were 2,5-dihydroxybenzoic acid (DHB) and α-cyano-4-hydroxycinnamic acid (CHCA), which were also evaluated in mixtures (1:1) and with the addition of sodium (NaCl solution, 0.1 M). The external calibrations were performed with flavonoid mixtures (quercetin, galangin, rutin and isoquercetrin). Pulsed ion extraction (PIE) of 100 ns, laser frequency of 1000 Hz and reflectron mode were applied in MS analyses. The mass spectra were averaged with 1000 shots. For MS/MS, the ions were accelerated to 19 kV in the LIFT cell.

### 3.4. MALDI and LDI Imaging

Mass spectrometry imaging (MSI) was performed on a MALDI-TOF/TOF UltrafleXtreme equipped with 1 KHz smartbeam II laser. The linear and positive modes were applied for protein analyses, while for secondary metabolites reflectron mode and both ionization methods (positive and negative) were used. The leaf cross-sections were generated using a Leica RM2245 microtome. Sections were adhered with double-sided tape (3 M Co., St. Paul, MN, USA) to indium tin oxide-coated conductive slides (Bruker Daltonics), analyzed without matrix by LDI (for flavonoids in LGT) using the follow parameters: 110 ns PIE, 1000 Hz laser frequency, reflector mode, 800 shots per position, minimum laser set and raster of 25 μm. For CGT analyses, the matrix (CHCA and DHB (1:1) at 10 mg/mL with the addition of 0.20 mg/mL NaCl) was applied to the tissue using the ImagePrep station with N_2_ flux and the matrix ions were used for internal calibration. These MSI experiments were performed with a raster of 35 μm. MSI for proteins was also determined using the previous parameters, with sinapinic acid (SA) at 20 mg/mL as the matrix. The images were performed in triplicate.

## 4. Conclusions

The results demonstrated the application and viability of MALDI imaging for analyzing proteins and secondary metabolites inside trichomes, achieving the optimized parameters to obtain informative and reliable images with high resolution. For the small trichomes that changed their shape and size with desiccation (a problem for the vacuum ion source in MALDI), several precautions are necessary, such the application of a proper matrix, size of the matrix crystals, laser set, isobaric ions, detector saturation, and the required resolution. The compounds, e.g., polymethoxylated flavonoids, can be ionized by LDI (without a matrix), to avoid some complications related to the presence of the MALDI matrix. However, non-conjugated compounds, such as sesquiterpenes and STL, require a matrix for ionization, which can be increased with the addition of sodium if the compounds show metal affinity (coordination reactions).

MSI of transversal leaf sections of *H. annuus* confirmed the distribution of polymethoxylated flavonoids on LGT (in leaf veins) and the sesquiterpene lactones on GCT (leaf intercostal areas). Therefore, determining the chemical composition of trichome types and their distribution among tissues are useful to infer the ecophysiological roles of metabolites.

## Supplementary Materials

The following materials are available online. Figure S1: Chromatogram at 210 nm of LGT); Figure S2: MALDI-MS image reconstructed from the ions *m*/*z* 403.17 and 401.16 [M + Na]^+^ corresponding to argophyllin B and argophyllone B (**A**), respectively, and the mass spectrum from MSI highlighting these ions; Figure S3: LDI-MS image reconstructed from ions *m*/*z* 329.06, 343.08, 359.08 and 373.09 [M − H]^−^, highlighting LGT (the arrow indicates the analyzed LGT); Table S1: Identification of chromatographic peaks of linear trichomes and UV, MS and MS/MS data.
