# Supplementary material for: Direct Analyses of Secondary Metabolites by Mass Spectrometry Imaging (MSI) from Sunflower (Helianthus annuus L.) Trichomes
Source: Molecules. 2017 May 10;22(5):774. doi: 10.3390/molecules22050774 (PMC6154581; doi:10.3390/molecules22050774)
Supplement: Supplementary file 1 [file molecules-22-00774-s001.pdf]

SUPPLEMENTARY MATERIAL

Perspectives and challenges for direct analyses of proteins and secondary metabolites by mass spectrometry imaging (MSI) as pictured on sunflower trichomes

Denise Brentan Silva,[1,2] Anna-Katharina Aschenbrenner,[3] Norberto Peporine Lopes,[2] Otmar Spring[3,*]

[1]Laboratório de Produtos Naturais e Espectrometria de Massas (LaPNEM), Universidade Federal de Mato Grosso do Sul, Campo Grande, MS, Brazil; denise.brentan@ufms.br

[2]Núcleo de Pesquisas em Produtos Naturais e Sintéticos (NPPNS), Faculdade de Ciências Farmacêuticas de Ribeirão Preto, Universidade de São Paulo, Ribeirão Preto, SP, Brazil; npelopes@fcfrp.usp.br

[3]Institute of Botany, University of Hohenheim, Garbenstraße 30, 70593 Stuttgart, Germany;katharina.aschenbrenner@uni-hohenheim.de; O.Spring@uni-hohenheim.de

*Correspondence: npelopes@fcfrp.usp.br (NPL), Fax: + 55 16 3602 4252.

Academic Editor: name
Received: date; Accepted: date; Published: date

1.1. UPLC-DAD-MS analyses of linear glandular trichomes

The LGT from central rib vein were manually collected, extracted with acetonitrile and diluted with water (9:1) for injection on UPLC-DAD-MS (the method is described in the manuscript- item 3.1). The chromatographic profile was illustrated in **Figure 1S** and the constituent data were summarized on **Table 1S**.

The metabolite identification was performed by injection of authentic standard (xanthomicrol) or from spectroscopic data (UV, MS and MS^2) data reported in the literature for *H. annuus* trichomes [14].

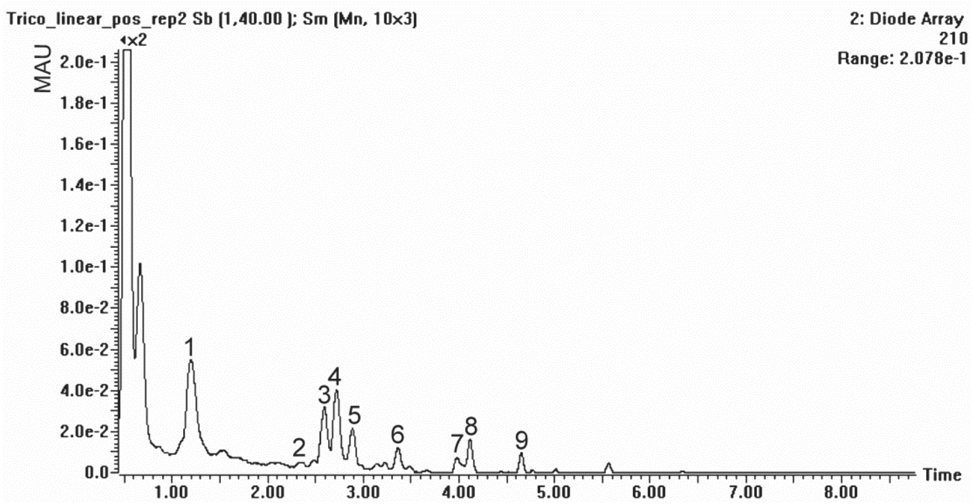

Figure S1. Chromatogram (at wavelength 210 nm) of an extract of sunflower linear glandular trichomes (LGT). For peak assignment see Table S1.

Table S1. Compound identification of chromatographic peaks (Figure S1) from the extract of sunflower linear trichomes and UV, MS and MS/MS data.

Peak	RT (min)	Compound	UV (nm)	Negative Mode (*m/z*)		Positive Mode (*m/z*)	
				MS $[M-H]^-$	MS/MS	MS $[M+H]^+$	MS/MS (eV)
1	1.30	Helibisabonol A	255, 280	267	-	269	-
2	2.49	Glandulone A	264	-	-	249	-
		Glandulone F		-	-	267	-
3	2.60	Demethoxysudachitin	269, 333	329	329 (20 eV)→314, 299, 240, 181, 143<br>329 (30 eV)→314, 299, 285, 279, 271, 268, 227, 211, 200, 179, 171, 145, 138, 117	331	331 (20 eV)→316, 301, 298, 273, 119<br>331 (30 eV)→316, 301, 285, 273, 241, 213, 181, 166, 155, 119
4	2.72	Acerosin	263, 334	359	359 (20 eV)→344, 329, 311, 298, 252, 213, 186, 119	361	361 (20 eV)→346, 331, 328<br>361 (30 eV)→346, 331, 328, 316, 301, 244, 227, 216, 193, 171, 151, 124<br>331 (20 eV)→316, 301, 271, 166, 109
		Glandulone D	263	-	-	249	
5	2.88	Helibisabonol C/heliannuol A/ heliannuol D	290	-	-	251	251 (15 eV)→175, 163, 153, 127, 109, 71
6	3.35	Sideritiflavone	283	359	359 (20 eV)→344, 329, 311	361	361 (20 eV)→346, 331, 328, 313, 197, 135
7	3.97	Nevadensin	263, 336	343	343 (20 eV)→328, 313, 298	345	345 (20 eV)→330, 315, 297, 266, 227, 194<br>345 (30 eV)→315, 297, 284, 272, 227, 183, 155
8	4.12	Xanthomicrol	263, 333	343	343 (20 eV)→328, 313, 298	345	345 (20 eV)→330, 315, 297, 266, 227, 194<br>345 (30 eV)→315, 297, 284, 272, 227, 197, 179, 151, 119
		Methylsudachitin	281, 332	373	373 (20 eV)→358, 343, 328<br>343 (25 eV)→328, 313, 285, 241, 167, 152	375	375 (20 eV)→360, 345, 315<br>345 (20 eV)→330, 315, 284, 267, 213, 183, 135
9	4.65	Glandulone E	256	-	-	249	249 (15 eV)→231, 193, 163, 151, 109, 71

RT: retention time.

1.2. MALDI-MS analyses

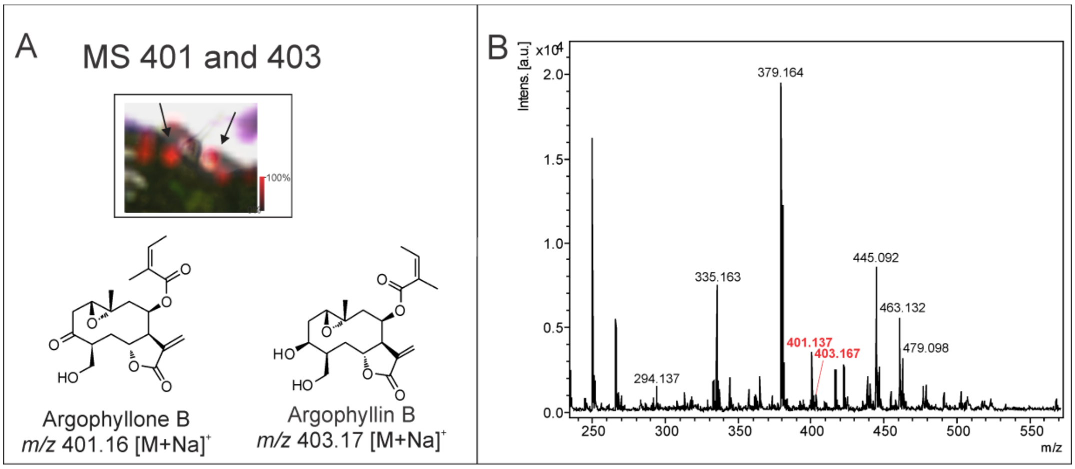

Figure S2. MALDI-MS image reconstructed from the ions *m/z* 403.17 and 401.16 $[M+Na]^+$ corresponding to argophyllin B and argophyllone B (**A**), respectively, and the mass spectrum from MSI highlighting these ions.

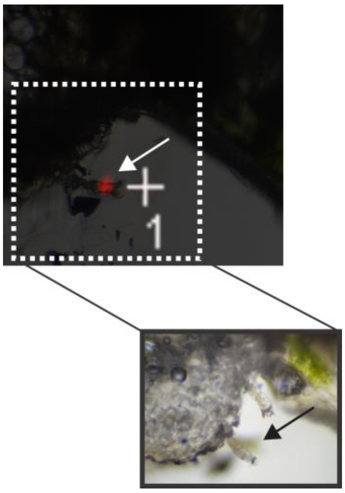

Figure S3. LDI-MS image reconstructed from ions *m/z* 329.06 (from demethoxysudachitin), 343.08 (nevadensin/xanthomicrol), 359.08 (sideritiflavone) and 373.09 $[M-H]^-$(methylsudachitin), highlighting the linear glandular trichome (LGT). The arrow indicates the LGT analyzed.
